# The associations of life course adversity on cognitive function and decline in the United States’ representative US Health and Retirement Study

**DOI:** 10.1002/alz70861_108363

**Published:** 2025-12-23

**Authors:** Carson Wong, Eli Puterman, Jordan Weiss

**Affiliations:** ^1^ University of British Columbia, Vancouver, BC Canada; ^2^ NYU Grossman School of Medicine, New York, NY USA

## Abstract

**Background:**

As the proportion of the US population aged 65 and over increases from 16.8% in 2020 to an estimated 22.8% by 2050, understanding how experiences over the life course shape trajectories of healthy aging becomes increasingly important. Maintenance of cognitive function is a key facet of healthy aging and may determine an individual’s ability to remain autonomous in later adulthood. Additionally, the growing social and economic burden of cognitive impairment makes it a vital public health concern and highlights the need to understand its risk factors.

**Method:**

We used 12 waves of data (1998–2020) from the Health and Retirement Study, a nationally representative longitudinal cohort of US adults over the age of 50 (n = 8,503). Cognitive function was assessed via a modified Telephone Interview for Cognitive Status. Life course adversity was derived from self‐reported childhood and adulthood economic and psychosocial exposures. Nonlinear mixed‐effects models evaluated associations between adversity and cognitive function, testing critical period, accumulation, and interaction hypotheses. Models were adjusted for race/ethnicity, sex, education, marital status, smoking, chronic conditions, alcohol use, and physical activity.

**Results:**

Greater childhood adversity was independently associated with lower baseline cognitive function (β = –0.74; 95% CI, –0.91 to –0.57; *p* < .001), consistent with a critical period effect. Adulthood adversity alone was not significantly associated with baseline cognition or decline. However, interaction models revealed that the adverse effect of adulthood adversity on cognitive decline was amplified among individuals with high childhood adversity (3‐way interaction: β = –0.014; 95% CI, –0.020 to –0.009; *p* < .001), suggesting compounding effects across the life span.

**Conclusion:**

Childhood adversity exerts a persistent influence on later‐life cognitive function. Adulthood adversity further accelerates decline, but primarily among those with early‐life disadvantage. These findings support both critical period and interaction models of life course risk and highlight the need for early preventive efforts and targeted interventions for high‐risk populations.

